# Overexpression of HE4 (human epididymis protein 4) enhances proliferation, invasion and metastasis of ovarian cancer

**DOI:** 10.18632/oncotarget.6327

**Published:** 2015-11-09

**Authors:** Liancheng Zhu, Huiyu Zhuang, Huimin Wang, Mingzi Tan, Carlton L. Schwab, Lu Deng, Jian Gao, Yingying Hao, Xiao Li, Song Gao, Juanjuan Liu, Bei Lin

**Affiliations:** ^1^ Department of Obstetrics and Gynecology, Shengjing Hospital Affiliated to China Medical University, Shenyang, Liaoning 110004, China; ^2^ Department of Obstetrics and Gynecology, Beijing Chaoyang Hospital Affiliated to Capital Medical University, Beijing 100043, China; ^3^ Department of Obstetrics, Gynecology and Reproductive Sciences, Yale University School of Medicine, New Haven, Connecticut 06520–8063, USA

**Keywords:** HE4, epithelial ovarian cancer, proliferation, invasion, metastasis

## Abstract

Overexpression of Human epididymis protein 4 (HE4) related with a role in ovarian cancer tumorigenesis while little is known about the molecular mechanism alteration by HE4 up regulation. Here we reported that overexpressed HE4 promoted ovarian cancer cells proliferation, invasion and metastasis. Furthermore, human whole genome gene expression profile microarrays revealed that 231 differentially expressed genes (DEGs) were altered in response to HE4, in which MAPK signaling, ECM receptor, cell cycle, steroid biosynthesis pathways were involved. The findings suggested that overexpressed HE4 played an important role in ovarian cancer progression and metastasis and that HE4 has the potential to serve as a novel therapeutic target for ovarian cancer.

## INTRODUCTION

Ovarian cancer is a kind of highly aggressive tumors associated with high mortality and morbidity in gynecology, it's the second cause of death among female reproductive malignancies and claims 140 200 lives each year [1]. Metastasis and invasion of early-stage ovarian cancer is a major factor responsible for its high mortality and poor prognosis [2]. Therefore, elucidating the molecular mechanisms underlying these changes will be important to facilitate the early diagnosis and treatment of ovarian cancer and to improve the prognosis of patients with this disease.

Human epididymis protein 4 (HE4), also known as whey-acidic-protein (WAP) four-disulfide core domain protein 2 (WFDC2), is a glycoprotein highly expressed in epithelial ovarian cancer (EOC) [3] and identified as a serum marker possessing higher sensitivity, specificity than CA125 in the confirmatory early diagnosis for EOC [4–6]. It shows better sensibility and specificity in the diagnosis of EOC recurrence with respect to CA125 and seems to be an independent predictive factor for the ideal tumor cytoreductive surgery and to maintain its prognostic role even after the recurrence [7]. In 2008, the FDA approved the use of HE4 assay for monitoring disease recurrence and therapeutic response in patients with epithelial ovarian cancer. More findings showed that serum HE4 during first-line chemotherapy could predict chemotherapy response [8], and it seems to be a good predictor of response and outcome in the neoadjuvant chemotherapy for those late stage high-grade serous ovarian cancer patients [9], and high HE4 serum levels correlated with chemoresistance and decreased survival rates in EOC patients [10, 11]. However, a host of researches are focusing on the clinical potential application of HE4 as a biomarker and predictor, little is known about the mechanism of its function, specifically the role of HE4 in the malignant biological behaviors of ovarian cancer. Recent studies showed that HE4 is associated with ovarian cancer cell adhesion, migration, proliferation, tumor growth and chemoresistance, which can be related to the activity of epidermal growth factor (EGF), vascular endothelial growth factor (VEGF) and insulin [10, 12, 13], these HE4–mediated invasion and metastasis may be promoted by its Lewis y fucosylation [14]. Nevertheless, a contrary result showed that HE4 might play a protective role by inhibiting cell proliferation in the progression of EOC [15], which makes the function of HE4 still unclear and controversial, the molecular mechanisms involved in HE4 need to be elucidated to provide insights about the biological processes modulated by it. Therefore, we aimed to investigate the alterations of malignant biological behaviors mediated by HE4 protein and its gene expression profile changes in response to the HE4 in ovarian cancer cells. The gene expression profile, especially the pathways generated from this investigation might lead to a better understanding about the molecular mechanisms associated with the HE4 in ovarian cancer.

## RESULTS

### Identification of HE4 gene transfection in ES-2 and Caov-3 cell lines

**Figure 1:**
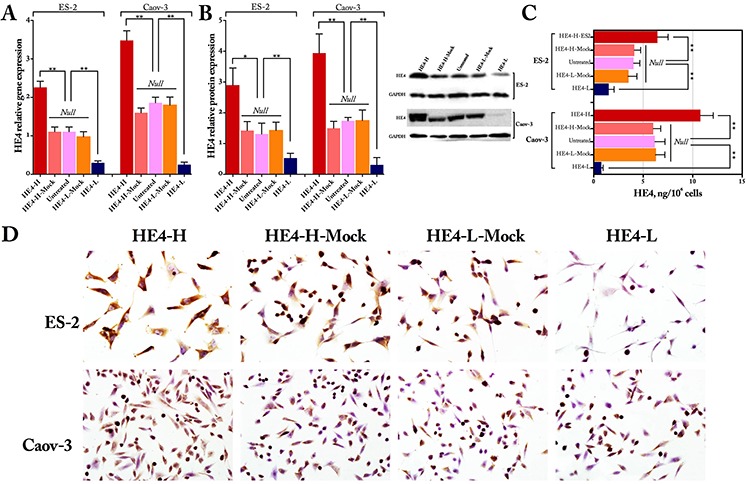
Identification of HE4 gene transfection Real time PCR results **(panel A)** immunoblots staining results **(panel B)** HE4 concentrations in conditioned media by ELISA **(panel C)** and immunocytochemistry images **(panel D)** showed the expression of HE4 after HE4 gene transfection (HE4-H, HE4 gene transfection; HE4-L, HE4 shRNA transfection) in ovarian cancer cell lines ES-2 and Caov-3, in which panels A and B are quantitative expressed as HE4 relative to GAPDH and the Western blotting image in panel B and ICC images in panel C are partly from Zhuang, H., et al., Mol Cancer, 2014. 13:p243.

Stable transfected cell lines were established on ES-2 and Caov-3 cell cells. The gene and protein expression levels of HE4 were obviously increased after HE4 transfection and decreased after the shRNA transfection, as detected by quantitative real-time PCR (Figure 1A) and western blot (Figure 1B), whereas there was no statistical difference in the protein expression of HE4 in the mock and untreated cells (Figure 1A, 1B). Moreover, similar alterations were also obtained by ELISA quantification of conditioned media (CM) for different cells, whereas the HE4 high expression groups maintained the highest secretory HE4 protein concentrations in CM and HE4 low expression groups maintained the lowest concentrations in CM, and there were no obvious change in the mock and untreated groups (Figure 1C, *P* < 0.01). Meanwhile, immunocytochemistry results were consistent with those of western blot analysis (Figure 1D). HE4 was detected as brown or yellow granules and localized predominantly in the cytoplasm of ovarian cancer cells, although membrane and peri-nuclear staining were also observed. All these results verified the HE4 gene transfection in ES-2 and Caov-3 cell lines.

### Overexpression of HE4 promotes ovarian cancer cell proliferation, invasion and metastasis

**Figure 2:**
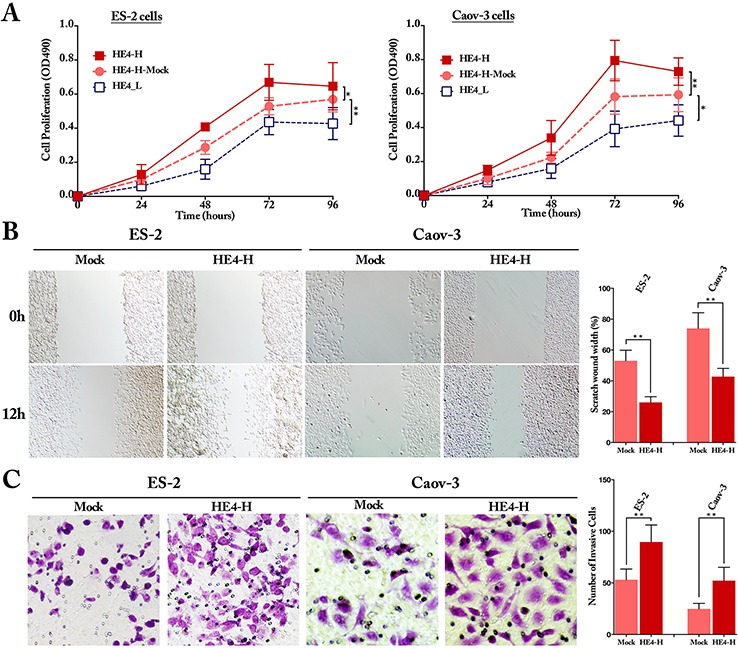
HE4 promoted the proliferation, invasion and metastasis capacities of ovarian cancer cells *in vitro* The proliferation assay showed that HE4 promoted the proliferation of the ovarian cancer cells ES-2 and Caov-3 obviously (**panel A,** *P* < 0.05), compared with the mock group. Wound healing assay indicated the invasion was enhanced obviously by HE4 in ovarian cancer cells ES-2 and Caov-3 (**panel B,** *P* < 0.01, original magnification 40). Transwell assay indicated the metastasis capacities were enhanced by HE4 in ovarian cancer cells ES-2 and Caov-3 (**panel C,** *P* < 0.01, original magnification 200).

To investigate the biological effects of HE4 on ovarian cancer cells *in vitro*, MTT, wound healing and transwell assays were performed in 2 types of ovarian cancer cells, ES-2 and Caov-3. MTT assay showed that compared with the mock cells, the proliferation ability of HE4-H cells was significantly enhanced whereas the HE4-L was obviously decreased both in ES-2 and Caov-3 cells (Figure 2A, *P* < 0.05). Wound healing and transwell assays showed that, compared with the mock groups, the high expression of HE4 significantly enhanced cell invasion and metastatic capacities (Figure 2B, 2C, *P* < 0.01). These results strongly suggest that overexpression of HE4 is able to promote the proliferation, invasion and metastasis of ovarian cancer cells *in vitro*.

**Figure 3:**
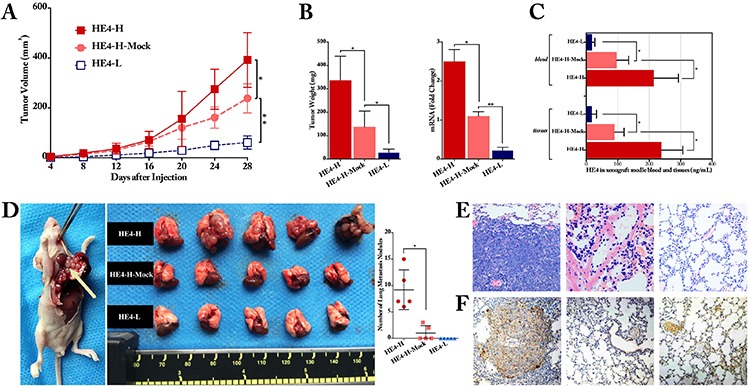
HE4 contributed to ovarian cancer cell progression and metastasis *in vivo* The xenograft tumor formation assay showed that, compared with the mock group, high-expressed HE4 enhanced tumor growth (*P* < 0.05), whereas low-expressed HE4 inhibited the tumor size formation significantly (*P* < 0.01, **panel A.**) At the end of the experiment, compared with the mock group, the mean tumor weight was remarkably heavier in the HE4-H group and lighter in HE4-L group (*P* < 0.05, **panel B).** The relative mRNA expression of HE4 in tumors was significantly higher in HE4-H group and lower in HE4-L group than the mock group (*P* < 0.01 and *P* < 0.05, respectively, **panel B.**). ELISA measurement revealed that HE4 protein level was obviously higher in the HE4-H group and lower in the HE4-L group than the mock group both in tumor tissues and blood of the nude mice (all *P* < 0.05, **panel C).** For metastasis assay, lung surface metastatic nodules (arrow in panel D.) after inoculation with cancer cells were observed and quantified, the metastatic nodules in HE4-H group were remarkable more than the mock group, and there was no nodule in HE4-L group **(panel D)** Representative H&E staining of lung tissues showed that metastatic tumor cells in lung tissues section **(panel E,** from left to right: HE4-H, HE4-H-Mock and HE4-L group). Immunohistochemical staining of HE4 for lung tissues further confirmed the H&E findings **(panel F,** from left to right: HE4-H, HE4-H-Mock and HE4-L group) the images in panel E and F are partly from Zhuang, H.,et al., Mol Cancer, 2014. 13:p243

Additionally, nude mouse xenograft model assays were conducted to confirm the role of HE4 *in vivo*. Xenograft tumor assay showed that, compared with the mock group, high expression of HE4 enhanced the tumor growth obviously, and low expression of HE4 significantly decreased the tumor formation (Figure 3A, *P* < 0.05). At the end of experiment, the mean tumor weight was 337.1 ± 102.2 mg, 137.8 ± 67.1 mg and 29.3 ± 13.2 mg in HE4-H, HE4-H-Mock and HE4-L group, respectively (*P* < 0.05, Figure 3B). The relative mRNA expression of HE4 in xenograft tumors was significantly higher in HE4-H group and lower in HE4-L group than the mock group (*P* < 0.01 and *P* < 0.05, respectively, Figure 3B). As to the protein concentration, HE4-H group obtained the most highest HE4 concentrations both in tumor tissues and blood by ELISA quantification, with values of 214.5 ± 78.2 ng/mL and 239.2 ± 67.5 ng/mL, moreover, HE4-L group gained the most lowest concentrations, with values of 18.2 ± 9.1 ng/mL and 17.5 ± 15.3 ng/mL, compared with the mock group 96.8 ± 37.8 ng/mL and 90.2 ± 29.8 ng/mL, respectively (*P* < 0.05, Figure 3C). These data reveal that HE4 promotes tumor formation obviously *in vivo*.

To investigate the role of HE4 in invasiveness and metastatic potential *in vivo*, 1 × 10^6^ HE4-H, Mock and HE4-L cells were injected into the tail vein of mice. 24 days later, an autopsy revealed a large number of lung metastatic nodules in the HE4 high expression group, few metastatic nodules were detected in the mock group, whereas almost no was observed in the low expression group (*P* < 0.01, Figure 3D). The results of H&E staining showed that the mice in all the high expression group were metastasized, whereas only 2 mice were metastasized among the mock group and there was no metastasis in the low expression group (Figure 3E). The metastasis rates in the HE4 high expression, mock and low expression groups were 100%, 40% and 0%, respectively (*P* < 0.01). Immunohistochemical staining confirmed the HE4 expression in HE4-H group, compared with the sparsely and no expression in Mock and HE4-L groups (Figure 3F). Taken together, both of the *in vitro* and *in vivo* results demonstrated that overexpression of HE4 promotes ovarian cancer cell proliferation, invasion and metastasis.

### Gene expression analysis and clustering

**Figure 4:**
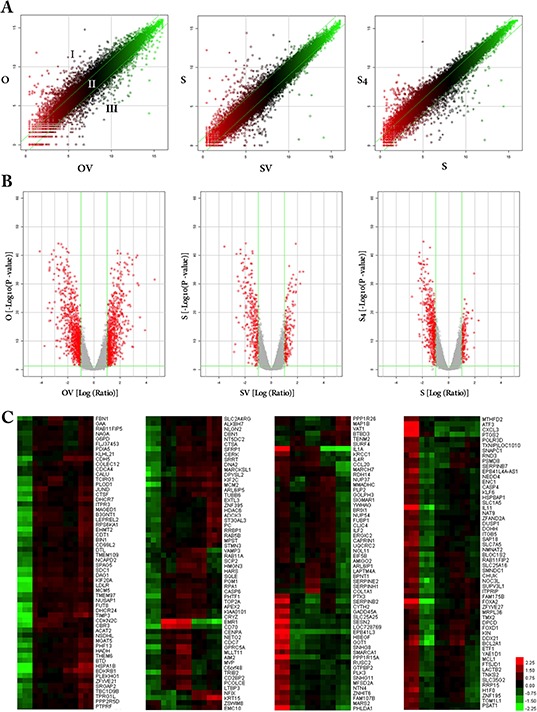
Scatter plots, volcano plots and hierarchical clustering map of DEGs in response to HE4 protein To visually assess the variation (or reproducibility) among microarrays, scatter plots **(panel A)** were performed, only probes with *P* < 0.05 were included. The central diagonal lines were used to classify gene expression levels into three groups (as shown in OV vs O): Group I, > 1-fold change increase in gene expression; Group II, gene expression levels within a 1-fold-change; and Group III, > 1-fold change decrease in gene expression. The volcano plots **(panel B)** showed the distribution of differentially expressed probes while line in green representing the cut-off, a measurement of fold-change on the x-axis versus a measure of significance (negative logarithm of the *P*-value) on the y-axis. The log scale of the expression signal values was plotted for all probes excluding control and flagged probes. So the red point in the plot represents the differentially expressed genes with statistical significance. Hierarchical clustering map was performed to visualize the correlations among the replicates and varying sample conditions **(panel C)** Up- and down-regulated genes are represented in red and green colors, respectively. A subset of differential genes was selected for clustering analysis. An intensity filter was used to select genes where the difference between the maximum and minimum intensity values exceeds 1500 among all microarrays. For this microarray project, the number of genes clustered was 231. The heatmap labels in each column from left to right are as follows: O1, O2, O3; OV1, OV2, OV3; SV1, SV2, SV3; S1, S2, S3; S4–1, S4–2, S4–3.

The expression profiles of all the samples passed the microarray quality control (Table 1), 3 scatter plots were constructed with a two-dimensional rectangular coordinate plane (Figure 4A). The volcano plots revealed the DEGs for each pair of gene chips, a total of 717 genes were up-regulated and 898 genes down-regulated in O vs OV, 166 genes up-regulated and 285 down-regulated in S vs SV, 164 genes up-regulated and 533 genes down-regulated in S4 vs S (Figure 4B). Using Hierarchical clustering map analysis with probe sets identified 231 DEGs in general (Figure 4C).

**Table 1:** The cell line samples description and RNA quality control

Sample ID	Label	OD260/280	OD260/230	Total Amount	RIN	Results
ES-2-HE4-H	O	2.02	2.21	16.10	9.30	Pass
ES-2-HE4-H-Vector	OV	2.02	2.35	12.10	8.60	Pass
ES-2-HE4-L	S	2.02	2.24	20.40	10.0	Pass
ES-2-HE4-L-Vector	SV	2.03	2.09	16.00	9.00	Pass
ES-2-HE4-L-Active	S4	2.07	1.82	13.00	6.10	Pass

### Validation of gene expression by quantitative RT-PCR

**Figure 5:**
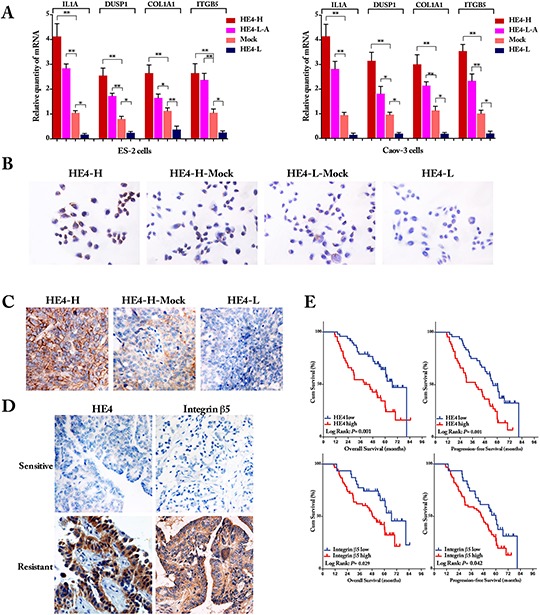
Validation of differentially expressed genes Quantitative real-time PCR for selected genes (IL1A, DUSP1, COL1A1 and ITGB5) found to be differentially expressed in gene microarrays. Both in ES-2 cells and Caov-3 cells, the relative mRNA expression of 4 genes was significantly higher in HE4 high expressed cells and lower in HE4 low expressed cells **(panel A,** compared with Mock cells, one-way ANOVA). ICC staining showed that integrin β5 was highly expressed in HE4-high expression cells, relatively low expressed in the mock cells (HE4-H-Mock and HE4-L-Mock) and sparsely expressed in HE4 low expression cells **(panel B)** Immunohistochemical staining in xenograft tumor tissues showed that integrin β5 was high expressed in all the HE4 highly expressed tumor tissues, relatively low expressed in the HE4-H-Mock tumors and totally not expressed in HE4-L tumors (100%, 80% and 0% in 3 groups, respectively, P=0.003, panel C). IHC staining of ovarian cancer sample showed that expressions of HE4 and integrin β5 were mainly on membrane and cytoplasm, their expressions were relatively lower in chemotherapy sensitive group than the resistant group, the correlation coefficient was 0.213 (Spearman correlation analysis, *P* = 0.042, panel D). Kaplan-Meier survival analysis **(panel E)** showed that high expressions of HE4 and integrin β5 were independent risk factors for overall survival (OS) and progression-free survival (PFS), log Rank *P* = 0.001, 0.001 for HE4 in OS and PFS, *P* = 0.029, 0.042 for integrin β5 in OS and PFS, respectively.

To validate the gene expression profile results, 4 DEGs (ie, IL1A, DUSP1, COL1A1 and ITGB5) were selected for quantitative RT-PCR analysis verification (Figure 5A). Generally, the trends for up or down regulation DEGs by quantitative real-time PCR analysis were consistent with those of the DEG expression profiling analysis, confirming the reliability of the microarray results.

### Validation of protein expression by ICC and IHC staining

To confirm gene expression results at the protein level, ICC staining for integrin β5 was applied in ES-2 cells, integrin β5 was highly expressed in HE4-high expression cells, relatively low expressed in the mock cells and sparsely expressed in HE4 low expression cells (Figure 5B).

The IHC high expression rates of integrin β5 in HE4 high expression, mock and low expression xenograft tumour tissues were 100%, 80% and 0%, respectively (Figure 5C). The HE4 high expression group displayed the highest integrin β5 staining. Furthermore, IHC staining for HE4 and integrin β5 were carried out on all paraffin embedded ovarian cancer samples. The expressions of HE4 and integrin β5 were mainly on membrane and cytoplasm (Figure 5D). There was significant difference in low and high expression between sensitive group and resistant group (all *P* < 0.01, Table 2). Spearman correlation analysis revealed that the expression of integrin β5 was positive linear related with the HE4 (*r* = 0.213, *P* = 0.042). However, no significant association was found between IHC expression and clinicopathological features of the patients (Table 3). Moreover, the high expression of HE4 and integrin β5 were good predictors for OS and PFS (Kaplan-Meier survival analysis, *P* < 0.05, Figure 5E), although multivariate COX analysis demonstrated that, together with FIGO stage III-IV, the high expression of HE4 was the independent factor for both of OS and PFS (*P* = 0.002, 0.001 for OS, PFS, respectively).

**Table 2:** Comparison of clinical characteristics and immunohistochemical expression of HE4 and Integrin *β5* in 92 cases of epithelial ovarian cancer

Characteristics	N	Group		*P*	HE4					Integrin β5				
		Sensitive	Resistant		−	+	++	+++	*P*	−	+	++	+++	*P*
**Age group, n(%)**														
≤60	74	47(83.9)	27(75.0)	0.292^‡^	16	17	17	13	0.065	13	24	19	7	0.161
>60	18	9(16.1)	9(25.0)		1	13	9	6		11	5	10	3	
**FIGO Stage, n(%)**														
I-II	31	27(48.2)	4(11.1)	<0.001^*‡^	10	7	9	5	0.081	11	8	8	4	0.435
III-IV	61	29(51.8)	32(88.9)		7	23	17	14		13	21	21	6	
**Differentiation, n(%)**														
Well	14	10(17.9)	4(11.1)	0.318^‡^	5	2	4	3	0.435	4	3	5	2	0.866
Moderate	43	28(50.0)	15(41.7)		8	15	13	7		12	12	15	4	
Poor	35	18(32.1)	17(47.2)		4	13	9	9		8	14	9	4	
**Pathological Subtype, n(%)**														
Serous carcinoma	60	36(64.3)	24(66.7)	0.815^‡^	8	22	16	14	0.251	17	16	19	8	0.460
Non-serous carcinoma	32	20(35.7)	12(33.3)		9	8	10	5		7	13	10	2	
**Lymph node metastasis, n(%)**														
No	63	43(76.8)	20(55.6)	0.032^*‡^	14	21	17	11	0.449	19	18	18	8	0.393
Yes	29	13(23.2)	16(44.4)		3	9	9	8		5	11	11	2	
**Residual tumor size, n(%)**														
≤1 cm	53	41(73.2)	12(33.3)	<0.001^*‡^	14	15	16	8	0.072	17	17	16	3	0.178
>1 cm	39	15(26.8)	24(66.7)		3	15	10	11		7	12	13	7	

* *P* < 0.05.

‡ Chi-square test.

**Table 3:** Expression of HE4 and integrin β5 in chemotherapy sensitive group and resistant group of 92 cases of epithelial ovarian cancer

Marker	Sensitive Group	Resistant Group	*P*^1^	r_s_^*^	P^*2^
	*n* = 56	*n* = 36			
**HE4**					
**−**	16	1	< 0.001	-	-
**+**	21	9			
++	14	12			
+++	5	14			
**Integrin β5**					
**−**	21	3	0.002	0.213	0.042
**+**	18	11			
++	15	14			
+++	2	8			

1 *P* value of Chi-square

2 *P* value of Spearman correlation compared with the expression of HE4.

* Correlated with the expression of HE4.

### GO function analysis and pathway result of differential genes

We used the GO analysis to group all these 231 DEGs in different groups that were predominantly involved in molecular function (MF), biological process (BP) and cellular component (CC), as shown in Table 4. Canonical pathway analysis demonstrated that a total of 10 pathways were enriched for the 231 DEGs (Table 5), such as MAPK signaling pathway, ECM-receptor interaction, steroid biosynthesis, cell cycle and CDK regulation of DNA replication.

**Table 4:** GO Enrichment Analysis for all the 231 DEGs

GO Term	Genes in Gene Set	Genes in overlap (k)	*P* value	FDR *q*-value
**molecular_function (Top 10)**				
DNA binding	602	15	2.80E-07	1.11E-04
Oxidoreductase activity	289	8	8.69E-05	1.72E-02
Nucleoside triphosphatase activity	212	6	5.96E-04	4.86E-02
Isomerase activity	35	3	6.41E-04	4.86E-02
Pyrophosphatase activity	226	6	8.32E-04	4.86E-02
Hydrolase activity acting on acid anhydrides	228	6	8.70E-04	4.86E-02
Histone deacetylase binding	10	2	1.01E-03	4.86E-02
Ubiquitin binding	11	2	1.23E-03	4.86E-02
Hydrolase activity acting on carbon nitrogen not peptidebonds	46	3	1.43E-03	4.86E-02
Low density lipoprotein binding	12	2	1.47E-03	4.86E-02
**biological_process (Top 10)**				
Programmed cell death	432	17	4.75E-11	2.89E-08
Cell development	577	19	6.99E-11	2.89E-08
Regulation of programmed cell death	342	15	1.52E-10	4.17E-08
Apoptosis GO	431	16	4.19E-10	8.63E-08
Regulation of developmental process	440	16	5.64E-10	9.31E-08
Regulation of apoptosis	341	14	1.48E-09	2.03E-07
Negative regulation of cellular process	646	15	6.78E-07	7.06E-05
Biopolymer metabolic process	1684	25	6.85E-07	7.06E-05
Negative regulation of programmed cell death	151	8	7.76E-07	7.11E-05
Negative regulation of biological process	677	15	1.21E-06	1.00E-04
**cellular_component (Top 10)**				
Cytoplasm	2131	46	7.97E-18	1.86E-15
Cytoplasmic part	1383	30	6.87E-12	8.01E-10
Intracellular organelle part	1192	24	4.33E-09	2.73E-07
Organelle part	1197	24	4.69E-09	2.73E-07
Nucleus	1430	26	7.62E-09	3.55E-07
Intracellular non membrane bound organelle	631	17	1.43E-08	4.75E-07
Non membrane bound organelle	631	17	1.43E-08	4.75E-07
Membrane	1994	29	1.25E-07	3.63E-06
Endoplasmic reticulum	294	11	2.22E-07	5.75E-06
Chromosome	124	7	2.53E-06	5.89E-05

**Table 5:** Canonical pathway analysis in the 231 DEGs

Pathway	Genes in Gene Set	Genes in Overlap	Gene Symbol	*P*-value	FDR
Steroid biosynthesis	17	4	*SQLE, DHCR7, NSDHL, DHCR24*	0.00000118	0.000475
DNA replication	36	4	*MCM2, MCM5, DNA2, RPA1*	0.0000272	0.0031
Lysosome	121	6	*GAA, CTSA, TCIRG1, CTSF, LAPTM4A, NAGA*	0.000028	0.0031
Endocytosis	183	7	*RAB11FIP5, RAB5B, RAB11FIP2, RAB11A, NEDD4, LDLR, HSPA1B*	0.0000321	0.0031
Cell cycle	128	6	*CDKN2C, MCM2, GADD45A, MCM5, CDC7, YWHAG*	0.0000385	0.0031
Glycosaminoglycan biosynthesis - keratan sulfate	15	3	*B3GNT1, ST3GAL3, FUT8*	0.0000478	0.00321
Lysine degradation	44	4	*PLOD1, ACAT2, HADH, EHMT2*	0.0000608	0.0035
CDK Regulation of DNA Replication	18	3	*MCM2, MCM5, CDT1*	0.0000849	0.00428
MAPK signaling pathway	267	7	*CHUK, GADD45A, IL1A, RPS6KA1, HSPA1B, DUSP1, JUND*	0.000334	0.0149
ECM-receptor interaction	84	4	*SDC1, ITGB5, DAG1, COL1A1*	0.000743	0.0299

### Interaction network for the DEGs in MAPK, ECM-receptor interaction and cell cycle pathways

**Figure 6:**
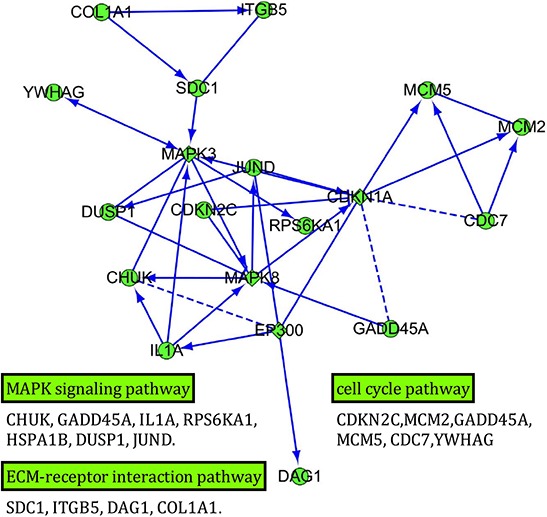
Interaction network of the differentially expressed genes in MAPK, ECM-receptor interaction and cell cycle pathways Genes with more links are shown in bigger size. The square represents the predicted genes. Arrow line represents definite control relationship, dotted line represents predicted control relationship, and solid line represents inhibition.

In order to further investigate the global expression occurring and to define how individual gene interacts with each other to have a coordinated role, we identified potential network for 3 pathways involved in the 231 DEGs, MAPK signaling pathway, ECM-receptor interaction pathway and cell cycle pathways (Figure 6). Among the interaction network diagram, a total of 4 interaction genes were predicted (MAPK3, MAPK8, EP300 CDKN1A), in which MAPK3 and MAPK8 seem to be the most connected predicted hub genes.

## DISCUSSION

Ovarian cancer has the highest mortality rate of all gynecologic cancers and up to 75% of diagnosed patients are already at an advanced stage. It is a neoplasm characterized by rapid growth, distinctive spreading, and formation of ascites. No definite symptoms related with early-stage disease and no effective screening methods make its early detection difficult. Meanwhile, the molecular mechanisms underlying ovarian cancer are poorly understood. In recent years, increasing attentions have been focusing on HE4 (Human Epididymis Protein 4) since its first shown to be highly expressed in ovarian cancer by cDNA microarrays in 1999 [16], especially on its clinical application as a predictor in ovarian cancer for early diagnosis [4], better tumor cytoreductive surgery [7], chemoresistance [8, 10] and prognosis [7, 10]. These increasing researches on HE4 clinical applications set off vigorously need to the basic mechanism of its function, unfortunately, the relevant work is far from enough. High-throughput technologies for assaying gene expression, such as high-density oligonucleotide and cDNA microarrays, may offer the potential to identify relevant genes highly associated with HE4 in ovarian cancer. Thus, this study showed the first communication of an investigation that involved the genome-wide examination of differences in gene expression profile alteration in response to HE4 in ovarian cancer cells.

In this study, we observed that HE4 protein enhanced the proliferation, invasion and metastasis of ovarian cancer cells obviously, and these biological behaviors are the major characteristics of the ovarian cancer [2, 17]. These findings are similar with the previous investigations on the biological effect of HE4 in EOC [10, 12] or endometrial cancer [18, 19], which validate the basic biological functions of HE4. However, it's still controversial as to the proliferation effect of HE4 on ovarian cancer. Our previous investigation [13] demonstrated that HE4 protein can enhance cell viability, promote accumulation in the G2/M phase in SKOV-3 cells after the recombinant HE4 protein stimulation. Similar effect was observed in the HE4 gene silencing experiment [20], which showed that the proliferation and tumor formation was obviously abolished by the knockdown of HE4 gene both *in vitro* and *in vivo* in SKOV3 cells. Our previous data [21] also demonstrated that the HE4 protein expression was significantly higher in malignant ovarian tissues compared to benign tumor and normal ovarian tissues, which revealed that the overexpression of HE4 enhanced the malignant extent of ovarian cancer.

To further explore the mechanisms underlying the effect of HE4 on the proliferation, invasion and metastasis of ovarian cancer cells, a well-designed gene expression profile analysis was applied to detect the gene alterations in response of HE4. Finally, 231 DEGs were identified, successive validation experiments were done to verify the creditability of microarray results, and interaction network diagram was performed according to pathway analysis of DEGs. A new study showed the DNA aptamers with affinity for HE4 by using capillary-based aptamer selection, high-throughput sequencing, and a freely available bioinformatics pipeline [22]. However, our investigation still seems to be the first report on the genome-wide gene expression profile alterations in response to HE4. The known functions of some genes can provide insights with the feasible function of HE4 in cancer, although others are still useful for a further research.

A recent study found that HE4 may play an important role in EGFR activation and the MAPK signaling pathway [12] in ovarian cancer cells, this result was further confirmed by findings that HE4 might inhibit cell proliferation by regulating MAPK and PI3K/AKT pathways *in vitro* [15] and HE4 was linked to the activity of EGF, VEGF, insulin and HIF1α [10]. In our study, by the use of high throughput and combinatorial gene expression profile analysis, we found for the first time that 7 genes out of 231 DEGs were involved in the MAPK signaling pathway in response to HE4 protein, which indicated that HE4 may exert its biological function such as enhancing proliferation, invasion and metastasis via MAPK pathway. However, tumor invasion and metastasis are a series of complex pathophysiological processes that include not only interactions between tumor cells and between tumor cells and host cells, but also a complex regulatory network involving multiple bioactive molecules [23]. Our data showed that, beside MAPK pathway, ECM-receptor interaction pathway also involved in the regulation of HE4 protein. Furthermore, our interaction network analysis merged these two pathways and predicted the potential hub genes. These analysis results illustrated that HE4 may promote the invasion and metastasis of ovarian cancer cells by activating MAPK or ECM-receptor pathways. Further investigations are still needed to focus on the roles of these credible potential relevant genes to elucidate the underlying basic mechanisms of HE4 in ovarian caner.

As a protein found in human epithelium of the distal epididymis, it is hardly surprising that our finding demonstrated that HE4 was involved in the steroid biosynthesis pathway. Until now, quite a few investigations are focusing on the interaction of HE4 and hormonal elements. A recent research demonstrated that 17b-estradiol, tamoxifen, and fulvestrant induce nuclear and nucleolar translocation of HE4 and that HE4 overexpression induces resistance to antiestrogens, resulted in estrogen receptor-α (ER-α) downregulation *in vitro* and in human ovarian cancers by interacting with it [24]. Together with our finding, it seems to have enough evidence to speculate the possible function of HE4 with steroid, and this can help the study for antiestrogen, which has been evaluated as chemotherapeutics for ovarian cancer, particularly in cases of platinum resistant disease.

Collectively, our data showed that HE4 promoted the proliferation, invasion and metastasis of ovarian cancer cells *in vitro* and *in vivo*, and our gene expression profile analysis identified 231 DEGs in response to HE4. HE4 may involve in the pathways such as MAPK signaling, ECM receptor, steroid biosynthesis pathway, etc. Elucidation of the molecular mechanism of HE4 may lead to the design of therapeutic strategies targeting HE4 for the treatment of ovarian cancer.

## MATERIALS AND METHODS

### Construction of expression vectors

Reverse transcription-PCR products produced from human cDNA and corresponding to full-length human HE4 was cloned in pGEX-4 T or pGEX-6 T vectors (Amersham Biosciences). An HE4 expression construct was generated by subcloning PCR-amplified full-length human HE4 cDNA into the pEGFP-N1 or pCMV6 plasmid. The following primers are used: P1: 5′-TCC GCT CGA GAT GCC TGC TTG TCG CCT AG-3′ and P2: 5′-ATG GGG TAC CGT GAA ATT GGG AGT GAC ACA GG-3′. Two shRNA expression vectors for human HE4 were constructed using the vector pSilence. The mRNA target sequences chosen for designing HE4-shRNA are GTC CTG TGT CAC TCC CAA T for HE4-shRNA1 and GAT GAA ATG CTG CCG CAA T for HE4-shRNA2.

### Cell culture and HE4 gene transfection

Human epithelial ovarian cancer cell lines ES-2 and Caov-3 were purchased from American Type Culture Collection (Manassas, VA, USA) and cultured in RPMI 1640 media with 10% FBS and antibiotics (penicillin-streptomycin, amphotericin B and tetracycline). Transfection was carried out using liposomes with a vector transfection kit according to the instructions. Stable cell lines over-expressing HE4, HE4 shRNAs cell lines and their respective empty-plasmid transfected cell lines were selected for 14 days with 800 ug/ml G418 (Invitrogen).

### Transfection identification by quantitative real-time PCR, western blot, ELISA and immunocytochemistry

#### Quantitative real-time polymerase chain reaction (RT-PCR)

Total mRNA was extracted from ovarian cancer cells using RNeasy Mini Kit (Qiagen, Valencia, CA, USA). cDNA was then synthesized by RNA reverse transcribing with a Super Script III First-Strand Synthesis System for RT-PCR Kit (Invitrogen). RT-PCR was performed on Roche LightCycler 480 (Roche Diagnostics, Mannheim, Germany) sequence detection system, using the following amplification conditions: 5 min, 95°C; followed by 40 cycles of 15 s 95°C, 1 min 60°C and 20 s 72°C. CT values were determined using the IQ5 software (Bio-Rad). The primers for HE4 were 5′- AGT GTC CTG GCC AGA TGA AAT G- 3′ for forward and 5′- CAG GTG GGC TGG AAC CAG AT- 3′ for reverse. The comparative threshold cycle method was used for the calculation of amplification fold, as specified by the manufacturer. The housekeeping gene glyceraldehyde-3-phosphate dehydrogenase (GAPDH) was used to normalize the quantity of complementary DNA that was used in the PCR reactions. The melting curves were analyzed after amplification. PCR reactions of each sample were done in triplicate. Data were analyzed through the comparative threshold cycle (CT) method.

#### Western blot

Total proteins extracts of each cell lines were resolved by 12% sodium dodecyl sulphate-polyacrylamide gel electrophoresis (SDS-PAGE), subjected to electrophoresis, and transferred onto a methanol activated polyvinylidene fluoride membrane overnight. After blocking, the membranes were washed four times with Tris-buffered saline containing 0.3% Tween-20 (TBST) at room temperature for 15 min and then incubated overnight at 4°C with HE4 antibody (Abcam, Rabbit polyclonal, 1:40). After washing, the membranes were incubated with HRP-conjugated secondary antibody (Santa Cruz) at room temperature for 1 h. The protein bands were visualized by Image J 1.31v and normalized relative to the GAPDH protein expression level.

#### Collection of conditioned media and ELISA assay

90% confluent cells were washed twice with PBS buffer and replenished with serum free DMEM media for 24 hours. HE4 protein concentrations of conditioned media (CM) were measured by ELISA assay following manufacturer's protocol (KA&M, Shanghai, China). Relative HE4 concentrations in the CM were normalized to the cell number in each well. Absorbance was measured at 450 nm on an MK3 microplate reader (Waltham, MA, United States).

#### Immunocytochemistry(ICC)

Cells at exponential phase of growth were digested by 0.25% trypsin and cultured in medium containing 10% FBS to prepare single-cell suspension. Cells were washed twice with cold PBS when growing in a single layer, and fixed with 4% paraformaldehyde for 30 min. SABC kit was used to detect the HE4 expression (1:300). The primary antibody was replaced by rabbit IgG for negative control. Images were obtained using a fluorescence microscope at magnification × 200.

### Measurement of cell malignant behaviors after HE4 transfection

#### Cell proliferation assay

Cell proliferation was assessed with the MTT (thiazolyl blue tetrazolium bromide) assay. Cells were plated in 96-well culture plates at 1 × 10^3^ cells per well containing 0.2 mL of culture media. After incubation for 24 h, 48 h, 72 h and 96 h, 0.02 mL of 5 mg/mL MTT was added to each well and incubated for 4 h at 37°C. The medium was then replaced with the stop solution (DMSO, 150 μL per well) and the absorbance at 490 nm was measured on a plate reader (EnSpire, Perkin Elmer Corporation, USA). The 0.1% DMSO controls were measured in parallel. Assays were repeated at least three times independently.

#### Wound healing assay

Cells were seeded at a density of 1 × 10^5^ cells/mL in six-well plates and incubated until 90% density. And then a scratch wound was made across the center of the monolayer of cells in each well by using a sterile 200 μL pipette tip. This was followed by incubation in medium without serum for 24 hours. Images of the cells that had migrated into the cell-free scratch wound area were acquired and the migration distance was measured under inverted microscope. The scratch wound widths were determined by the relative percentage compared to untreated control cells.

#### Transwell assay

The Matrigel were melted and put at 4°C refrigerator overnight the day before this experiment. The pipette tip was pre-cooled in ice-cold for 0.5 h during experiment, and the ECM gel was diluted by 1:8 with serum free medium, Matrigel 100 μL was added into the upper chambers, the whole process was performed on ice. Then they were placed in an incubator at 37°C for 5 h. 10^5^/mL cells in logarithmic growth phase was added in each well for 200 μL, 500 μL medium supplemented with 10% fetal bovine serum were added in lower chamber. After culturing for 24 h, nutrient solution was abandoned and a cotton swab was used to gently wipe out the upper layer of transwell. Membrane of transwell was fixed with methanol for 20 min, washed with PBS 3 times, and then staining with 0.1% crystal violet for 20 min after airing. The invasive cell numbers of 5 fields (upper and lower, left and right, middle) were counted under microscope, the mean value was obtained and the statistical analysis was made. The cells of each group were treated in triplicate and experiments were repeated three times.

#### Xenograft tumor assay

Female BALB/c nu/nu mice (4–6-weeks-old) were obtained from Shanghai Institute of Material Medicine (Shanghai, China) and maintained in specific pathogen-free conditions (temperature 23°C −25°C and humidity 40%-50%). All experimental protocols were approved by the Committee for the Care and Use of Laboratory Animals of Shengjing Hospital affiliated to China Medical University (Permit No: 2014PS163K) and all experiments were performed in accordance with the approved guidelines and regulations.

For the subcutaneous tumor model, ES-2 cells expressing high HE4, Mock and low HE4 at the exponential phase of growth were digested by 0.25% trypsin and washed with PBS and resuspended with physiological saline at a concentration of 1 × 10^5^ cells/μL. A volume of 0.1 mL (1 × 10^7^) suspended cells was subcutaneously injected into the right flank of anesthetized mice (*n* = 6 for each group) and the mice were observed for 4 weeks. The xenograft tumor volume was measured every 4 days and calculated as (W^2^ × L)/2, where W and L refer to the shorter and longer dimensions of the tumor, respectively. All the tumors were removed and weighted and then divided into 3 parts for following study: one of which was fixed with 4% paraformaldehyde for immunohistochemical staining, and the other two were snap-frozen in liquid nitrogen for detection of HE4 by quantitative realtime mRNA and ELISA methods. Blood samples were collected through cardiac puncture under CO_2_ anesthesia and then naturally coagulated for 20 min after collection. Samples were centrifuged at 1000 *g* for 10 min, and the supernatant was collected and preserved at −20°C.

For assessment of the influence of HE4 on metastasis *in vivo*, tail-vein injection experiments were performed on nu/nu mice as previously described [25]. A volume of 200 μl (1 × 10^6^) suspended cells was injected into the tail-vein of nu/nu mice (*n* = 5 for each group) and the mice were observed for 24 days. The mice were then killed humanely and an autopsy was performed and the lungs were examined for tumors. Then, tissues were dehydrated, processed, and embedded in paraffin wax. Serial sections 5-μm thick were prepared from each block, stained with haematoxylin and eosin (H&E) and analyzed by immunohistochemistry (HE4 antibody, 1:2000).

There were no animal deaths during the experiment and all tumor-implanted animals were humanely euthanized at the end of the experiment by overdose of pentobarbital (50 mg/kg; *ip*). The criteria for the humane endpoint were a tumor size > 10 mm in diameter and/or the presence of ulceration, necrosis, or infection. HE4 concentrations in the serum samples and the supernatants of tumor tissues of mice were measured by ELISA method shown above.

#### Total RNA extraction and gene chip hybridization

We randomly selected cell line ES-2 for gene expression profile analysis. 3 groups of cells were prepared for further analysis: ES-2-HE4-H vs ES-2-HE4-H-Vector, ES-2-HE4-L-Active (ES-2-HE4-L cells treated with exogenous active HE4 protein [Novoprotein, USA] 100 ng/ml for 12 hours) vs ES-2-HE4-L, and ES-2-HE4-L vs ES-2-HE4-L-Vector, all cells were labeled and listed in Table 1. Total RNA was extracted from all cell lines using RNeasy Mini kit (Qiagen, Valencia, CA, USA) and further purified with RNeasy Min-elute Clean-up Columns (Qiagen, Valencia, CA), as described by the manufacturers. The total RNA of each cell lines were distributed and detected in 3 chips to decrease the experimental error, thus a total of 15 microarrays was used to identify gene expression profiles. RNA quantity and purity was assessed using NanoDrop ND-1000. Pass criteria for absorbance ratios are established as A260/A280 ≥ 1.8 and A260/A230 ≥ 1.5 indicating acceptable RNA purity. RIN values are ascertained using Agilent RNA 6000 Nano assay to determine RNA integrity. Pass criteria for RIN value is established at ≥ 6 indicating acceptable RNA integrity.

Fluorescent aRNA targets were prepared from 1 or 2.5 μg total RNA samples using OneArray^®^ Amino Allyl aRNA Amplification Kit (Phalanx Biotech Group, Taiwan) and Cy5 dyes (Amersham Pharmacia, Piscataway, NJ, USA). Fluorescent targets were hybridized to the Human Whole Genome OneArray^®^ (HOA6.1) with Phalanx hybridization buffer using Phalanx Hybridization System. This array contains 30,275 DNA oligonucleotide probes, and each probe is a 60-mer designed in the sense direction. Among the probes, 29,187 probes correspond to the annotated genes in RefSeq v38 and Ensembl v56 database. Besides, 1,088 control probes are also included. After 16 hours hybridization at 50°C, non-specific binding targets were washed away by three different washing steps (Wash I 42°C 5 mins; Wash II 42°C, 5 mins, 25°C 5 mins; Wash III rinse 20 times), and the slides were dried by centrifugation and scanned by Axon 4000B scanner (Molecular Devices, Sunnyvale, CA, USA). The intensities of each probe were obtained by GenePix 4.1 software (Molecular Devices).

#### Data analysis and clustering

The raw intensity of each spot was loaded into Rosetta Resolver System^®^ (Rosetta Biosoftware) to process data analysis. The error model of Rosetta Resolver System^®^ could remove both systematic and random errors form the data. Those probes with background signals were filtered out. Probes that passed the criteria were normalized by 50% median scaling normalization method. The technical repeat data was tested by Pearson correlation coefficient calculation to check the reproducibility (R value > 0.975). Normalized spot intensities were transformed to gene expression log2 ratios between the control and treatment groups. The probes with log2 ratio ≥ 1 or log2 ratio ≤ −1 and *P*-value < 0.05 were defined as differential expression genes (DEGs) for further analysis. Scatter plots were made to visualizingly assess the variation between chips. A hierarchical clustering and volcano plots were performed to visualizingly show a distinguishable gene expression profiling among samples.

### Validation for gene expression by RT-PCR, ICC and immunohistochemistry

#### RT-PCR

Real-time polymerase chain reaction was performed in triplicate with primer sets and probes that were specific for 4 selected genes that were found to be significantly differentially expressed: IL1A, DUSP1, COL1A1 and ITGB5, both in ES-2 cells and CaoV3 cells. The methods are shown as above.

#### ICC

Immunocytochemistric method was done to detect the expression of integrin β5 that is among the DEGs in over-expressing HE4, HE4 shRNAs and the mock ovarian cancer cells ES-2. The concentration of integrin β5 monoclonal (Abcam, Rabbit) antibody was 1:50. The methods are shown as above.

#### Immunohistochemistry (IHC)

To further evaluate protein expression levels for DEGs, in view of forward investigation, immunohistochemical staining for HE4 and Integrin β5 were performed on ovarian tissue samples. In our previous studies, we have established a set of 92 cases of ovarian cancer paraffin embedded samples from Shengjing Hospital of China Medical University [11, 26], and we designed to detect the protein expression of HE4 and Integrin β5 in these samples and to analyze their correlation. According to criteria set forth in the 2012 NCCN (National Comprehensive Cancer Network) guidelines of chemotherapy resistant and sensitive, all these 92 patients were divided into 2 groups, in which 56 patients were considered sensitive (including 2 patients who were partially sensitive to chemotherapy) and 36 patients were in the resistant group. All the patients had undergone cytoreductive surgery of EOC. The general clinical and pathological information of patients were shown in Table 2. Rabbit polyclonal anti-HE4 antibody and rabbit polyclonal anti-integrin β5 antibody were purchased from Abcam Company (USA). The staining procedure was performed as described in the manuals for the SABC (Streptavidin-Biotin Complex) and SP (streptavidin-peroxidase) kits. The working dilutions of HE4 antibody and integrin β5 antibody were 1:50 and 1:200, respectively. Negative controls were performed by omission of the primary antibody or incubation with an isotype control antibody. Positive controls were performed as follows: a normal epididymis tissue sample for HE4 and a normal small intestine tissue sample for Integrin β 5. The presence of brown colored granules on the cell membrane or in the cytoplasm was taken as a positive signal, and was classified according to color intensity as follows: not colored, light yellow, brown, and tan which were recorded as 0, 1, 2, and 3, respectively. A positive cell rate of less than 5% was 0, 5~25% was 1, 26~50% was 2, 51~75% was 3 and more than 75% was 4. The final score was determined by multiplying the positive cell rate and score values: 0~2 was considered negative (−), 3~4 was (+), 5~8 was (++), and 9~12 was (+++). – and + was considered as low expression, ++ and +++ as high expression. Two observers read the sections to control error. Survival analysis was performed on those patients, and the overall survival (OS) and progression-free survival (PFS) time was defined from the date of surgery (earliest was in July, 2004) to the date of death, occurrence or the last follow-up (May, 2015). Meanwhile, the expression of integrin β 5 in xenograft tumors was assessed.

### Enrichment analysis of DEGs

All the DEGs that passed the cluster analysis were prepared to run Gene Ontology (GO) analysis and canonical pathways analysis (KEGG and Biocarta). The gene regulatory network was visualized by Cytoscape [27], proteins in the network served as the nodes, and each pairwise protein interaction (referred to as edge) was represented by an undirected link. The property of the network was analyzed with the plug-in network analysis.

### Statistics

Statistical analyses were performed using SPSS program (Version 22 for Mac; SPSS Inc., Chicago, IL, USA) and the graphs were completed using Graphpad Prism 6.0e Software for Mac OS X (GraphPad Software, La Jolla California USA, http://www.graphpad.com). Student's *t*-test was employed for comparison between two groups, Chi-square and one-way ANOVA with LSD or Bonferroni post hoc test was used for comparison between more than two groups. Quantitative data are presented as Mean ± SD. As to the analysis of quantitative RT-PCR result, data were expressed as mean ± SEM to compare on mRNA expression between different groups. Survival analysis was analyzed using Kaplan- Meier curves, and significant differences between groups and among different immune-markers were tested using the log-rank test. *P* value of < 0.05 was considered statistically significant, in figures, *, *P* < 0.05, **, *P* < 0.01.
